# The influence of mode of remote delivery on health-related quality of life outcome measures in British Sign Language: a mixed methods pilot randomised crossover trial

**DOI:** 10.1007/s11136-024-03864-0

**Published:** 2024-12-11

**Authors:** Katherine D. Rogers, Antonia Marsden, Alys Young, Chris Evans

**Affiliations:** 1 Social Research with Deaf People (SORD), Division of Nursing, Midwifery and Social Work, School of Health Sciences, Faculty of Biology, Medicine and Health, University of Manchester, Oxford Road, Manchester, M13 9PL UK; 2 Division of Population Health, Health Services Research & Primary Care, School of Health Sciences, Faculty of Biology, Medicine and Health, University of Manchester, Manchester, UK; 3 Centre for Deaf Studies, University of the Witwatersrand, Johannesburg, South Africa; 4 Grupo de Investigación Bienestar, Universidad de Las Américas, Quito, Ecuador

**Keywords:** Outcome assessment, Sign language, Deaf populations, Telemedicine

## Abstract

**Objectives:**

Little is known about the efficacy of remotely delivered outcome measures (psychological/health-related assessments) in a signed language for Deaf people. The objective is to explore the equivalence of two modes of remote delivery of health-related quality of life outcome measures in British Sign Language (BSL): asynchronous online assessment versus synchronous live face-to-face online assessment in sign language.

**Methods:**

Thirty-one participants were recruited through Deaf networks and sign language media. Measures used were validated BSL versions of the EQ-5D-5L, EQ-VAS and CORE-10. A randomised, crossover trial was conducted between March and May 2023 with seventeen first receiving asynchronous assessment and sixteen first receiving synchronous live online assessment. This study explored whether the outcomes of the two assessments are equivalent regardless of modality of delivery. Demographic data were collected, and eight participants took part in semi-structured qualitative interviews exploring modality preferences and the impact of each modality.

**Results:**

The mean difference between pre-recorded and live modes was 0.034 for EQ-5D-5L BSL (90% CI 0.015–0.051), 4.33 mean difference for EQ-VAS BSL (90% CI 0.697–8.083), and mean difference of 0.17 for CORE-10 BSL (90% CI − 1.4065 to 1.1775). The confidence intervals for each of the EQ-5D-5L BSL, EQ-VAS BSL, and CORE-10 BSL lie within the prespecified equivalence margins which suggested that the two modes are equivalent.

**Conclusion:**

The results demonstrate that EQ-5D-5L BSL, EQ-VAS BSL, and CORE-10 BSL may be considered as equivalent across modes of remote delivery. This further strengthens the validation of existing standardised assessments in BSL.

A BSL version of the abstract is available in Supplementary Video 1.

**Supplementary Information:**

The online version contains supplementary material available at 10.1007/s11136-024-03864-0.

## Plain English summary

Little is known about psychological and health-related assessments delivered online in a signed language for Deaf people. The aim is to see if a pre-recorded online assessment completed independently are equal (or not) to live face-to-face online assessment in British Sign Language (BSL). Thirty-one participants were recruited to the study through Deaf networks and sign language media. BSL versions of the EQ-5D-5L, EQ-VAS and CORE-10 were used. Between March and May 2023, a randomised crossover trial was done with seventeen participants completing the online pre-recorded assessment, first and sixteen completing the live online assessment, first. The aim was to explore whether the results of the two assessments were the same regardless of whether they were pre-recorded or live. Information about the participants was collected, and eight participants took part in semi-structured interviews to ask about preferences and the impact of each assessment. The results show that the two different ways of doing the assessments produced similar results and may be considered equivalent. This also strengthens the BSL versions of the pre-existing assessments.

## Introduction

### Background

Deaf Sign language users often identify themselves as being culturally Deaf and are part of the Deaf community, seeing their language use as a source of identity not an adaptation to hearing loss. Signed languages are visual languages, grammatically distinct from spoken languages; in the UK, British Sign Language (BSL) [1]. A systematic review on health outcomes of Deaf signing populations worldwide has highlighted inequalities in both mental and physical health outcomes for Deaf people in comparison with general populations [2]. For example, Deaf people experience poorer health status overall [3], higher rates of depression/anxiety [4, 5], higher rates of obesity [6] and, in one study, were diagnosed at a more advanced stage of colorectal and prostate cancer [7] than the general population. In the UK, the context for this study, Deaf people experience inequities in accessing services compared to hearing populations [8] resulting in under- and late- diagnoses, poor management of chronic conditions and reduced healthcare choices. Accessible Deaf-specialist sign language primary mental/physical health services are not universally available in the UK. A key barrier in service delivery is the scarcity of healthcare professionals with the expertise and cultural competency to work with Deaf patients [9]. Consequently, Deaf patients living in areas without such professionals with the required expertise are more likely to experience healthcare inequity. In such circumstances, telemedicine has been proposed as a potential way forward to increase the availability of services in signed languages whether directly or through interpreters.

In the UK, the National Health Service (NHS) defines telemedicine as “the use of telecommunication and information technology for the purpose of providing remote health assessments and therapeutic interventions” [10]. For Deaf people this can include live interactions in a signed language such as remote psychological therapy [11], the provision of static information such as health literacy through videos in signed languages [12], and standardised assessments of health/psychological health in a signed language. These can be pre-recorded asynchronous assessments in signed languages [4, 13–16] or directly delivered assessments by a professional who signs to a Deaf patient/service user in an online clinical session without an interpreter. While patients are strongly recommended to use online portals to complete assessments before their sessions with a clinician [17], there are no clear guidelines for those patients who are Deaf sign language users. In response to the Covid-19 pandemic, services were rapidly forced to accept this medium to deliver clinical, health and social care services which accelerated their use in mainstream services. Findings from the systematic review carried out by Rogers et al. [18] on the effectiveness of telemedicine intervention with Deaf people suggested that delivery of mental health services using telemedicine is feasible and that satisfaction is high (e.g. [11, 19]) but the impact of different modalities of delivery on outcomes is under researched.’Modality’ refers to both medium of delivery e.g. online, or in real life as well as to linguistic modality (spoken, written, signed). It also encompasses features of response such as asynchronous self-completion or synchronous live interaction.

In general population studies, a meta-analytic review examined the equivalence of scores between paper-and-pencil and computer administration when completing Patient-Reported Outcome Measures (PROMs) concluding they were equivalent [20]. However, no similar studies on effects of modality of administration and response have been carried out with regard to signed languages.

The objective of this pilot study was to explore the equivalence of two modes of remote delivery of health-related quality of life outcome measures (EQ-5D-5L BSL, EQ-VAS BSL, and CORE-10 BSL): in asynchronous pre-recorded assessment in BSL and synchronous, live, delivery of the same assessment in BSL (via videoconferencing). The intention was to use the data to determine the sample size required for a confirmatory study, should one be needed.

## Methods

The study protocol for this study has been pre-registration and is available on the OSF (see Study One https://osf.io/7g2hu).

### Design

This is a randomised crossover experimental study with a subsequent nested qualitative exploratory study to assist with interpretation of results. A randomised crossover design was chosen for this study instead of the more traditional randomised parallel-group design because it was important to evaluate both modalities using the same participant. It required fewer participants than a parallel trial as each participant acted as their own control, and the risk of a carry-over effect was small. Participants were randomly allocated to one of two sequences of assessment. Mode A followed by Mode B (Group one = AB), or Mode B followed by Mode A (Group two = BA). Mode A delivered the assessment online, asynchronously, in BSL using pre-recorded test items, derived from the BSL test versions that had previously been tested for reliability and validity [15, 21]. Mode B also delivered the assessment online, but live. Random allocation was done in blocks (block sizes of 2 and 4) through https://www.sealedenvelope.com/. This block randomisation was carried out to ensure an equal size for each group. A sub-sample of the assessed group took part in individual semi-structured qualitative interviews to explore responses to the two modalities of assessment delivery and response that had been experienced in order to gather data to assist with the interpretation of the results of the experimental study.

### Sample

**Fig. 1:**
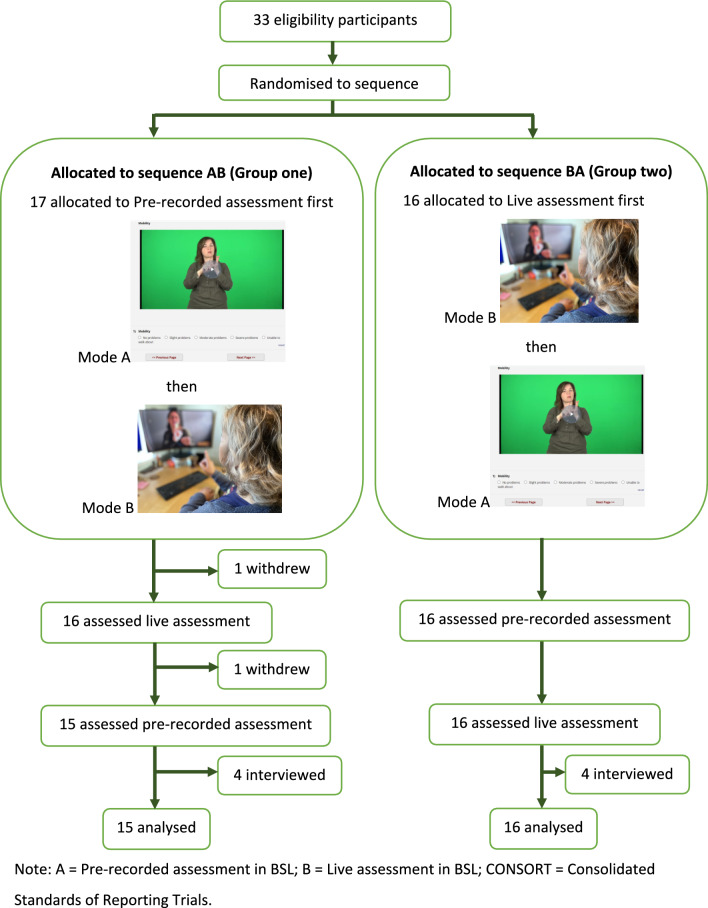
CONSORT flow diagram for crossover trial. *Note*: A = Pre-recorded assessment in BSL; B = Live assessment in BSL; CONSORT = Consolidated Standards of Reporting Trials

Deaf people who use BSL in the UK general population were recruited in various ways including snowball sampling, mail outs, social media and adverts on the project website. All recruitment materials were in BSL (and written English). The inclusion criteria stated participants needed to be Deaf, BSL users, and aged 18 years-or above. A sample size of 30 was chosen based on the recommended rule of thumb to estimate a parameter in Lancaster et al. [22]. This sample size enables the determination of equivalence where the equivalence margin has a Cohen’s d effect size of 0.83 with 80% power and a 5% significance level. Additional power comes from the crossover study design. The data from this study are intended to be used to determine the sample size required for a confirmatory study, should one be needed. Thirty-three Deaf BSL users initially took part completing the first round of assessments but subsequently two withdrew, leaving a total number of thirty-one participants who completed all aspects of data collection (see Fig. 1).

### Procedure

The study received ethical approval from the University of Manchester Research Ethics Committee (ref. 2022–14080-23435). Following individual consent and randomisation, participants, were informed whether allocated to Group one (AB) or two (BA). The assessor, a Deaf signer (Author 1), was different from the Deaf signer on the asynchronous version. She was also the original designer of the BSL validated assessments used in Mode A. The live-signed test items (Mode B) were identical to those in the asynchronous version (Mode A). In neither Mode was an interpreter used. It was assumed that there would be little or no change in responses to the assessment over the one to two weeks between delivery Modes. Demographic information collected included age, gender, hearing status of parents, as well as a self-report of current difficulties (if any) with their physical health and/or mental health (see Table 1). The rules for completing the BSL assessments for both conditions were the same: question repetition was allowed but giving specific examples of what a question meant was not permitted. In Mode A, repetition meant the participant rewatched the recorded question again. In Mode B, the researcher signed the question again.

**Table 1:** Demographic information of the study participants (N = 31)

	Group 1 (AB)	Group 2 (BA)	TOTAL
	n (%)	n (%)	N (%)
Age in years			
30–39	2 (13.3%)	4 (25%)	6 (19.4%)
40–49	8 (53.3%)	8 (50%)	16 (51.6%)
50–64	1 (6.7%)	2 (12.5%)	3 (9.7%)
+ 65	4 (26.7%)	2 (12.5%)	6 (19.4%)
Mean (SD)	52 (13.79)	47 (11.97)	49 (12.9)
Gender identity			
Female	8 (53.3%)	9 (56.3%)	17 (54.8%)
Male	7 (46.7%)	6 (37.5%)	13 (41.9%)
Transwoman	0 (0%)	1 (6.3%)	1 (3.2%)
Ethnicity			
Asian/Asian British Chinese	0 (0%)	1 (6.3%)	1 (3.2%)
Asian/Asian British Pakistani	1 (1%)	0 (0%)	1 (3.2%)
Mixed/Multiple ethnic group: Black Caribbean and White	0 (0%)	1 (6.3%)	1 (3.2%)
Mixed/Multiple group: Any other	0 (0%)	1 (6.3%)	1 (3.2%)
White: English/Welsh/Scottish/Northern Irish/British	14 (93.3%)	13 (81.3%)	27 (87.1%)
Sexuality			
Gay man	3 (20%)	3 (18.8%)	6 (19.4%)
Gay woman/lesbian	1 (6.7%)	2 (12.5%)	3 (9.7%)
Straight/heterosexual	10 (66.7%)	10 (62.5%)	20 (64.5%)
Pansexual	0 (0%)	1 (6.3%)	1 (3.2%)
Prefer not to say	1 (6.7%)	0 (0%)	1 (3.2%)
Consider disabled			
Yes	14 (93.3%)	10 (62.5%)	24 (77.4%)
No	1 (6.7%)	5 (31.3%)	6 (19.4%)
Prefer not to say	0 (%)	1 (6.3%)	1 (3.2%)
Employment status			
Yes	9 (60%)	12 (75%)	21 (67.7%)
No	6 (40%)	4 (25%)	10 (32.3%)
Currently experiencing mental health difficulties			
Yes	8 (53.3%)	8 (50%)	16 (51.6%)
No	6 (40%)	6 (37.5%)	12 (38.7%)
I don’t know	1 (6.7%)	1 (6.3%)	2 (6.5%)
Missing answer	0 (0%)	1 (6.3%)	1 (3.2%)

A purposively sampled sub-group of participants was invited to take part in a one-to-one semi-structured interviews to discuss their responses to the different modes of assessments in BSL. Diversity in sample selection was sought to cover a range of age, gender and sexuality with equal numbers in groups one and two. The aim of the interview was to explore preferences for mode of delivery having experienced both and why one might be more effective than another in their eyes. These data were designed to assist in the interpretation of the quantitative data, rather than to be a standalone qualitative study. Nine participants were invited, 8 responded and 8 were interviewed. Interviews were carried out online via Zoom in BSL by the researcher who carried out the assessment in Mode B and recorded with consent. Participants were sent in advance the topics that would be covered in the interview to permit them time to think prior to interview. These were presented as open questions covering: overall appraisal; pros of Mode A, pros of Mode B, cons of Mode A, cons of Mode B, additional or unexpected reflections. Interviews lasted an average of 9 min (range 6.5 to 15 min).

### Measures

The equivalence of three outcome measures across two delivery modes was investigated: (i) EQ-5D-5L (BSL version), (ii) EQ-VAS (BSL version), and (iii) CORE-10 (BSL version). The EQ-5D-5L contains five dimensions of health (mobility, self-care, usual activities, pain/discomfort and anxiety/depression) and includes the standard EQ-VAS which asks the participants to rate their health on that day from 0 (‘the worst health state you can imagine’) to 100 (the best health state you can imagine’). The EQ-5D-5L index score, also known as utility value, ranges from 0 (representing death) to 1 (representing perfect health) and is commonly used in health economics to measure the valuation of health states. The reliability and validity of the EQ-5D-5L BSL are reported in the study by Rogers et al. [15]. The short version of the Clinical Outcomes in Routine Evaluation-Outcome Measure (CORE-10) has ten items from the full version of CORE-OM that measures psychological distress [23]. The BSL version of the CORE-OM has been checked for its reliability and validity [21]. The CORE-6D is not a standalone measure but is a scoring based on six of the CORE-OM items to create a health utility scaling [24] which, like the EQ-5D ranges from a possible zero to 100. Whereas four of the five domains of the EQ-5D-5L are physical and the one (anxiety/depression) is psychological, only one of the six CORE-6D items, “I have been able to do most things I needed to” is arguably physical as well as psychological. Although the scores of the EQ-5D-5L and the CORE-6D encompass both quality of life/health utility scores, they have different foci. In this study, by permission of the CORE authors, the 14 items common to the CORE-10 and the CORE-6D scoring were presented without the other items of the CORE-OM respecting the greater time needed for BSL presentations of questionnaires. The focus of the qualitative semi-structured interviews are provided in the procedure section.

### Analysis

#### Statistical analysis

Descriptive analyses and the crossover analysis were run in SPSS Version 28. An equivalence testing approach was taken [25, 26]. In detail: (i) the difference between the first and second EQ-5D-5L BSL, EQ-VAS BSL and CORE-10 BSL scores for each participant were calculated; (ii) two-sample t-tests were carried out to compare the mean period difference in those in randomised group one (AB) and those in randomised in group two (BA) (mean period difference D); and (iii) the point estimate of the difference in outcomes between the two modes were calculated by D/2. A test for a carry-over effect was performed [27]. An equivalence limit of − 0.08 to 0.08 for the EQ-5D-5L index scores and − 10.00 to 10.00 for the EQ-VAS scores have been reported as Minimally Important Differences (MID)s in previous studies [28]. No such prior intervals have been proposed for the CORE-10 or CORE-6D hence potential equivalence limits were pre-determined using the methods of Machin et al. [29]. This method defines an equivalence region for the paired difference in mean scores of outcomes as (− delta, + delta) where delta is 20% of the overall observed mean. The recruitment and attrition rates were carefully monitored, means and standard deviations for outcome measures were used to calculate the sample size for a powered study.

The convergent validity of the EQ-5D-5L BSL, was assed using the CORE-10 BSL for the psychopathology correlates and the CORE-6D BSL for the health economic correlates. Although the convergent validity of the EQ-5D-5L with CORE-10 BSL has been reported in a previous study [15] which used a pre-recorded mode, convergent validity for the live mode had yet to be examined.

#### Qualitative analysis

Semi-structured interviews were analysed using the qualitative description approach which collates participant comments against pre-set questions but does not claim to be inductive or thematic [30, 31]. This was because the interviews were designed to serve a particular purpose to assist with the interpretation of the quantitative data rather than to be an exploratory phenomenological or ethnographic study. Author 1 grouped participant responses under pre-determined headings (overall appraisal; pros of Mode A, pros of Mode B, cons of Mode A, cons of Mode B, additional or unexpected reflections). No specialist qualitative search and retrieve software was used because of the highly structured nature of the questions asked mirroring the categories of analysis and the purpose of the qualitative component of the research design not requiring a more inductive approach. These were reviewed by Author 3 to highlight points of convergence or difference within individual participant responses (e.g. when pros and cons were opposite, or approaches to modality inconsistent) as well as between participants where wider themes emerged e.g. cultural suitability, insecurity regardless of mode. Illustrative quotes in the results section are English translations of the original BSL.

## Results

### Demographic information of participants

The mean age of participants was 49 years old (SD = 12.9; range: 31–76 years old). At least half of the participants were female, and majority (87.1%) were White British. Eleven had at least one Deaf parent, of whom ten use BSL. Sixteen participants reported currently experiencing mental health difficulties. All participants used BSL with 83.9% listing it as their ‘preferred’ language, 3 (9.7%) preferred Sign Supported English (SSE), and 2 (6.5%) preferred speech/lip-reading. All were happy to take the assessments in BSL. See Table 1 for the demographic information.

### Score equivalence

Table 2 shows the mean difference score and equivalence bounds for EQ-5D-5L BSL, EQ-VAS BSL, and CORE-10 BSL. The mean score for EQ-5D-5L BSL live and EQ-5D-5L BSL pre-recorded were similar (mean difference = 0.034 with 90% confidence interval of 0.015–0.051) and the EQ-VAS score 0.697 to 8.083. Both lie within the acceptable equivalence limit of − 0.08 to 0.08 for EQ-5D-5L index scores and equivalence bounds of − 10.00 to 1.00 for the EQ-VAS [28] (see Table 2). This suggests that the two modes of live and pre-recorded EQ-5D-5L BSL and EQ-VAS BSL assessments are equivalent.

**Table 2:** Mean difference scores and equivalence bounds for both modes of remote delivery of EQ-5D-5L, EQ-VAS, and CORE-10 assessments in BSL

	Pre-recorded Mean (SD)	Live Mean (SD)	Mean difference	Midpoint between mean live and pre-recorded	Equivalence bounds reported in Wang et al. [28]	90% CI for mean difference
EQ-5D-5L BSL Index score	0.848 (0.15)	0.814 (0.16)	0.034	0.8307	(− 0.08, 0.08)	(0.015, 0.051)
EQ-VAS BSL score	77.10 (15.65)	72.77 (16.04)	4.33	74.935	(− 10, 10)	(0.697, 8.083)

	Pre-recorded Mean (SD)	Live Mean (SD)	Mean difference	Midpoint between mean live and pre-recorded	Delta 20%/equivalence bounds	90% CI for mean difference
CORE-10 BSL score	10.06 (4.59)	10.23 (4.45)	0.17	10.145	(− 2.029, 2.029)	(− 1.407, 1.178)

**Fig. 2:**
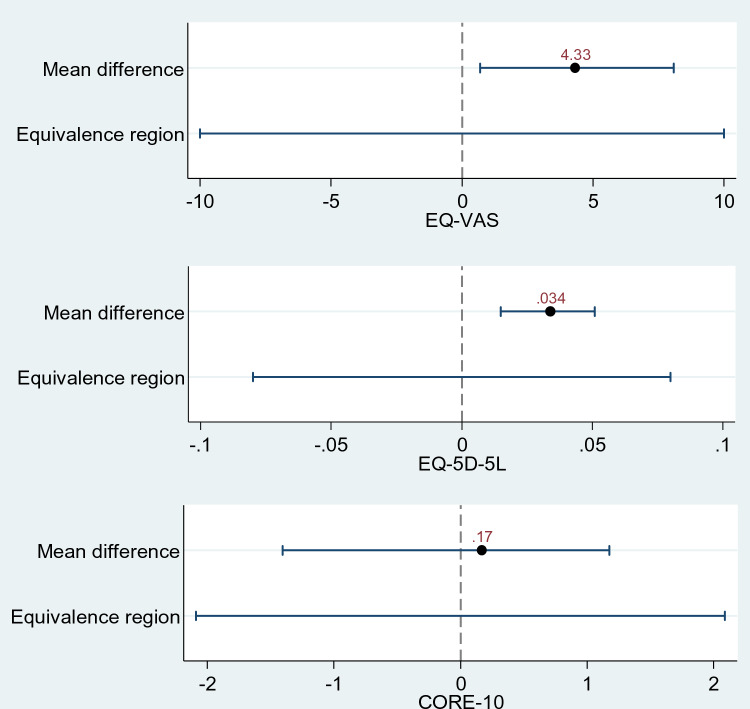
Equivalence margins and 90% confidence intervals for EQ-5D-5L BSL, EQ-VAS BSL, and CORE-10 BSL

The mean difference between the CORE-10 BSL live and CORE-10 BSL pre-recorded is 0.17, and the midpoint across mean live and pre-recorded is 10.145. Therefore, the delta 20% is 2.029, meaning the equivalence bounds are (− 2.029, 2.029). The 90% CIs (− 1.4065, 1.1775) for mean differences lies within this equivalence region (− 2.029, 2.029) which suggest the two modes are equivalent (see Fig. 2 for the equivalence margin and 90% confidence intervals for CORE-10 BSL).

### Utility values

The mean score of utility values for the EQ-5D-5L BSL and CORE-6D BSL for two modes are reported in Table 3. The mean scores are similar for both modes (mean utility values of 0.848 for EQ-5D-5L BSL (SD = 0.149) and 0.843 for CORE-6D BSL (0.116) for pre-recorded versions; and 0.814 for the live EQ-5D-5L BSL (SD = 0.160) and 0.856 for the live CORE-6D BSL (SD = 0.098).

**Table 3:** Utility values as measured by EQ-5D-5L BSL and CORE-6D BSL for pre-recorded and live modes

	Pre-recorded		Live	
	Mean	SD	Mean	SD
EQ-5D-5L BSL index values	0.848	0.149	0.814	0.160
CORE-6D BSL	0.843	0.116	0.856	0.098

For the pre-recorded mode, the Pearson’s correlation coefficient between EQ-5D-5L BSL utility value and CORE-6D BSL utility value is 0.650 which is significant (*r* = 0.650, n = 31, *p* < 0.001). A significant association was also found for the live mode between EQ-5D-5L BSL utility value and CORE-6D BSL utility value (r = 0.655, n = 31, p < 0.001).

### Convergent validity

Positive Pearson’s correlation was found between EQ-5D-5L BSL live and CORE-10 BSL live (*r* = − 0.408, n = 31, *p* < 0.023), and between EQ-5D-5L BSL live and CORE-6D BSL live (*r* = -0.586, n = 31, *p* < 0.001). For the pre-recorded mode, positive correlation was also reported for between EQ-5D-5L BSL pre-recorded and CORE-10 BSL pre-recorded and between EQ-5D-5L BSL pre-recorded and CORE-6D BSL pre-recorded (*r* = − 0.546, n = 31, *p* < 0.002 and *r* = − 0.714, n = 31, *p* < 0.001).

### Post hoc sample size calculations for a suitably powered study

Additional sample size calculations for each of the outcomes for the EQ-5D-5L BSL, EQ-VAS BSL and CORE-10 BSL were calculated with the data provided. For each, this was determined as the sample size required for a paired t-test to detect equivalence within the pre-specified equivalence limits based on the observed standard deviation of the differences in outcomes between the two modes of delivery, with 90% power and a 5% significance level. The sample sizes determined were N = 7, N = 12 and N = 28 for EQ-5D-5L BSL, EQ-VAS BSL and CORE-10 BSL respectively. These are very low as the observed differences between the modes of delivery were small. It was determined that the sample size of 30 was adequate to address the aims of this study.

### Findings from the interviews

Participants’ demographic details are provided in Supplementary Table 1. All thought that both modes of assessment in BSL were clear, easy to understand and felt able to answer the questions. Neither mode was seen as disadvantaging them, but different pros and cons for each were identified as discussed below. No one mode emerged as preferable, however when asked to make a clear choice it was how safe the style of assessment made them feel, rather than mode of delivery per se that made a difference. Two participants reported a preference for pre-recorded with the main reasons being that they felt safer and less self-conscious when answering the questions. Four participants preferred the live mode as it felt more natural, allowed them to interact, and they felt safer because if they felt vulnerable, they could seek support. Two participants stated that they did not have a preference and thought both would be fine.

#### Mode A

Participants reported similar advantages: (i) being able to replay the video if wanted; (ii) able to see the options for the response scale for each question on the screen all the time; (iii) reduced feelings of being conscious about being watched in relation to their answers; (iv) can complete the questionnaire at any time and at their own pace.

Good pace, and I thought it allowed me enough time to answer. I could see the options button clearly which allowed me to answer easily. [ID135]

The cons were more particular to individual participants, rather than shared by the whole group. They included: (i) the lack of live interaction. This led one participant to feel like they were talking to a robot and another wondering that might not have been assessed properly and another wondering if they had been fully understood.

I prefer live mode. But it doesn’t mean that it has to be in person as it can be done remotely. I prefer a real person, not pre-recorded version. …. With the pre-recorded version, it doesn’t feel human enough, which leaves me with the feeling that I have not been assessed properly. It feels like I have been seen, but left me feeling not confident that I have been understood. [ID111]

Some were concerned that if there was no live interaction then someone might be left feeling distressed and unable to ask for support. (ii) linguistic concerns. One was concerned about being unable to ask for clarification if unsure about a specific sign being used—the example given was the sign for ‘humiliated’ which does not include any lip pattern or fingerspelling. Another felt the BSL register used in this mode was too simple although another reported concerns that those with a low level of BSL may struggle to understand the pre-recorded version. (iii) that it could be time consuming. One participant commented they had to wait for each video to be completed, including viewing the answer options before being able to answer the questions.

#### Mode B

Pros: (i) Some of the pros reported by participants referred to their thoughts about what an ideal live assessment might provide rather than necessarily what they had experienced. For example, one participant suggested the live mode was advantageous because it was possible to ask for clarification, especially when a regional sign was used as sometimes there is no one universal sign despite this option not being possible under the test conditions of the research. Another commented that the live mode meant that the person who is asking the questions could adjust their BSL register depending on the person’s level of BSL; also disallowed under research conditions. (ii) several participants felt that the live mode was more natural and more engaging, and more akin to Deaf culture because of the constant eye contact and facial responsiveness that is part of the language; (iii) a few remarked that it was easier for someone who is not technically confident or competent with a computer; (iv) one participant pointed out that because of the sensitivity of the questions, you can say that you do not feel comfortable rather than not answering; (v) the same person concerned about lack of support under pre-recorded mode suggested it is possible to ask for help or support straight away when doing the assessment or immediately afterwards if the questions brings up difficult feelings.

… with live mode you could ask for support if you needed to. [ID110]

The cons were: (i) the live mode could be more difficult as participants had to remember what each of the five response options were although they were given at each question every time; (ii) some said they felt more self-conscious, uncomfortable, and exposed when answering the question to someone live; (iii) with the Deaf community being so small, one person said they worried about the potential loss of confidentiality with the person who is asking the questions; (iv) time could be a problem for two reasons. For some, the live mode meant that there was less flexibility about when they did the assessment. For others the live mode felt like they were under pressure to answer immediately rather than take time. (v) although the assessment did not mean they had to explain their answers, some felt compelled to do so to another Deaf person.

[in live mode] … sometimes I felt the temptation to explain the reason for each of my answers. With pre-recorded, I cannot do that. I think it is a Deaf culture thing to feel the need to explain each of my answers. [ID 106]

## Discussion

This is the first study to report on whether two modes of an assessment delivered remotely in a signed language are equivalent. Equivalence was found between pre-recorded and live assessments in BSL when delivered online for all three assessments. This suggests that online assessment in BSL, whether asynchronous (pre-recorded) or synchronous (live) likely does not interfere with how a Deaf person responds to a standardised assessment. The results are likely to be generalisable to other self-report outcome measures in other signed languages internationally. With the high prevalence of mental health difficulties and health inequalities in Deaf signing populations [2], the availability of standardised assessments in the relevant national signed language is vital, providing these assessments have been validated to use with Deaf people. The equivalence demonstrated for asynchronous, online assessment has positive implications for cost-effectiveness and convenience.

No strong participant preference for mode of delivery was evident but some issues that cut across modality emerged as important including concerns about safety and support, fit with Deaf cultural norms of interaction and technical challenges in online assessment. No influence was discerned in the order of administration between modes suggesting that personal preferences played little part in the overall outcomes of the assessments.

The utility values were explored in both modes for the EQ-5D-5L BSL and CORE-6D BSL, and the association between two measures were found to be significant. This indicates that the utility values for health states which have been validated in previous studies [15], can be used with signing Deaf populations in the UK. The EQ-5D-5L is a generic measure that can be used for assessing the outcomes of interventions, whereas the CORE-6D might be more suitable for mental health [24].

This study was initially intended as a pilot study, but sample size calculations based on the data collected for this study suggest the sample size was adequate to address the aims. Nonetheless, we suggest caution when interpreting the results and would welcome a further study to confirm the results. The implications should not be generalised to other assessments, such as the BSL Cognitive Screening Test [32], which require the presence of an assessment administrator. One limitation is in real-world practice, patients are likely to ask for examples to clarify the questions; explicitly disallowed in this study. Additionally, the researcher who signed in Mode B also conducted the interviews, potentially introducing a social desirability bias. The sample is limited by lack of diversity in ethnicity. The qualitative component is limited by its purpose to assist in the explanation of the quantitative results rather than to explore in depth the experience of the participants. Consequently, common validation techniques such as dual coding or participant checking were not conducted. A further limitation of this study is that the self-selecting participants might be more confident users of digital and communication technologies than others in the Deaf community.

## Conclusion

The findings suggest that the EQ-5D-5L BSL, EQ-VAS BSL, and CORE-10 BSL are equivalent which further strengthens the validation of the existing standardised assessment in BSL. Additionally, the results show that both modes of delivery may be considered as equivalent thus potentially allowing greater accessibility to standardised assessments in BSL for Deaf people and promoting the greater use of telemedicine for this purpose.

## Supplementary Information

Below is the link to the electronic supplementary material.

Supplementary file1 (MP4 72882 KB)

Supplementary file2 (DOCX 27 KB)

## Data Availability

The data supporting the findings reported in this paper are openly available from the figshare repository at https://doi.org/10.48420/25780404.v1.

## References

[1] Ladd, P. (2003). *Understanding deaf culture: In search of deafhood*. Multilingual Matters.

[2] Rogers, K. D., Rowlandson, A., Harkness, J., Shields, G., & Young, A. (2024). Health outcomes in deaf signing populations: A systematic review. *PLoS ONE,**19*(4), e0298479. 10.1371/journal.pone.0298479 38625906 10.1371/journal.pone.0298479 PMC11020444

[3] Shields, G. E., Rogers, K. D., Young, A., Dedotsi, S., & Davies, L. M. (2020). Health state values of deaf British Sign Language (BSL) users in the UK: An application of the BSL version of the EQ-5D-5L. *Applied Health Economics and Health Policy,**18*(4), 547–556. 10.1007/s40258-019-00546-8 31942693 10.1007/s40258-019-00546-8

[4] Rogers, K. D., Young, A., Lovell, K., Campbell, M., Scott, P. R., & Kendal, S. (2013). The British sign language versions of the patient health questionnaire, the generalized anxiety disorder 7-item scale, and the work and social adjustment scale. *Journal of Deaf Studies and Deaf Education,**18*(1), 110–122. 10.1093/deafed/ens040 23197315 10.1093/deafed/ens040 PMC3521778

[5] Kushalnagar, P., Reesman, J., Holcomb, T., & Ryan, C. (2019). Prevalence of anxiety or depression diagnosis in deaf adults. *The Journal of Deaf Studies and Deaf Education,**24*(4), 378–385. 10.1093/deafed/enz017 31369098 10.1093/deafed/enz017 PMC6786504

[6] Barnett, S., Klein, J. D., Pollard, R. Q., Samar, V., Schlehofer, D., Starr, M., Sutter, E., Yang, H., & Pearson, T. A. (2011). Community participatory research with Deaf Sign Language users to identify health inequities. *American Journal of Public Health,**101*(12), 2235–2238. 10.2105/AJPH.2011.300247 22021296 10.2105/AJPH.2011.300247 PMC3222424

[7] Druel, V., Hayet, H., Esman, L., Clavel, M., & Bugat, M. E. R. (2018). Assessment of cancers’ diagnostic stage in a Deaf community—Survey about 4363 Deaf patients recorded in French units. *BMC Cancer,**18*(1), 93. 10.1186/s12885-017-3972-3 29361910 10.1186/s12885-017-3972-3 PMC5781319

[8] Alexander, A., Ladd, P., & Powell, S. (2012). Deafness might damage your health. *The Lancet (British Edition),**379*(9820), 979–981. 10.1016/S0140-6736(11)61670-X 10.1016/S0140-6736(11)61670-X 22423872

[9] Hulme, C., Young, A., Rogers, K., & Munro, K. J. (2023). Cultural competence in NHS hearing aid clinics: A mixed-methods case study of services for Deaf British sign language users in the UK. *BMC Health Services Research,**23*(1), 1440. 10.1186/s12913-023-10339-4 38114981 10.1186/s12913-023-10339-4 PMC10731837

[10] NHS Data Model and Dictionary. (2023). *Telemedicine*. [cited July 17, 2023]. Available from: https://www.datadictionary.nhs.uk/nhs_business_definitions/telemedicine

[11] Crowe, T., Jani, S., Jani, S., Jani, N., & Jani, R. (2016). A pilot program in rural telepsychiatry for deaf and hard of hearing populations. *Heliyon,**2*(3), e00077. 10.1016/j.heliyon.2016.e00077 27441259 10.1016/j.heliyon.2016.e00077 PMC4946006

[12] McKee, M. M., Hauser, P. C., Champlin, S., Paasche-Orlow, M., Wyse, K., Cuculick, J., Buis, L. R., Plegue, M., Sen, A., & Fetters, M. D. (2019). Deaf adults’ health literacy and access to health information: Protocol for a multicenter mixed methods study. *JMIR Research Protocols,**8*(10), e14889. 10.2196/14889 31599730 10.2196/14889 PMC6812478

[13] Crowe, T. V. (2002). Translation of the Rosenberg Self-Esteem Scale into American Sign Language: A principal components analysis. *Social Work Research,**26*(1), 57–63. 10.1093/swr/26.1.57

[14] Fellinger, J., Holzinger, D., Dobner, U., Gerich, J., Lehner, R., Lenz, G., & Goldberg, D. (2005). An innovative and reliable way of measuring health-related quality of life and mental distress in the deaf community. *Social Psychiatry and Psychiatric Epidemiology,**40*(3), 245–250. 10.1007/s00127-005-0862-9 15742231 10.1007/s00127-005-0862-9

[15] Rogers, K. D., Pilling, M., Davies, L., Belk, R., Nassimi-Green, C., & Young, A. (2016). Translation, validity and reliability of the British Sign Language (BSL) version of the EQ-5D-5L. *Quality of Life Research,**25*(7), 1825–1834. 10.1007/s11136-016-1235-4 26887955 10.1007/s11136-016-1235-4 PMC4893373

[16] Kushalnagar, P., Paludneviciene, R., Kallen, M., Atcherson, S., & Cella, D. (2020). PROMIS-deaf profile measure: Cultural adaptation and psychometric validation in American sign language. *Journal of Patient-Reported Outcomes,**4*(1), 44. 10.1186/s41687-020-00208-7 32519000 10.1186/s41687-020-00208-7 PMC7283401

[17] The National Collaborating Centre for Mental Health. (2024). *NHS Talking Therapies for anxiety and depression Manual. Version number 7*. The National Collaborating Centre for Mental Health; March 2024. [cited 2024 Sept 6]. Available from: https://www.england.nhs.uk/wp-content/uploads/2018/06/NHS-talking-therapies-manual-v7-1.pdf

[18] Rogers, K., Lovell, K., & Young, A. (2023). What is the efficacy and effectiveness of telemedicine intervention for deaf signing populations in comparison to face-to-face interventions? A systematic review. *BMC Health Services Research,**23*(1), 678. 10.1186/s12913-023-09509-1 37349811 10.1186/s12913-023-09509-1 PMC10288820

[19] Pertz, P., Plegue, M., Diehl, K., Zazove, P., & McKee, M. (2018). Addressing mental health needs for deaf patients through an integrated health care model. *Journal of Deaf Studies and Deaf Education,**23*(3), 240–248. 10.1093/deafed/eny002 29562357 10.1093/deafed/eny002

[20] Gwaltney, C. J., Shields, A. L., & Shiffman, S. (2008). Equivalence of electronic and paper-and-pencil administration of patient-reported outcome measures: A meta-analytic review. *Value Health,**11*(2), 322–333. 10.1111/j.1524-4733.2007.00231.x 18380645 10.1111/j.1524-4733.2007.00231.x

[21] Rogers, K., Evans, C., Campbell, M., Young, A., & Lovell, K. (2014). The reliability of British Sign Language and English versions of the Clinical Outcomes in Routine Evaluation-Outcome Measure with d/Deaf populations in the UK: An initial study. *Health and Social Care in the Community,**22*(3), 278–289. 10.1111/hsc.12078 24206212 10.1111/hsc.12078

[22] Lancaster, G., Dodd, S., & Paula, R. W. (2004). Design and analysis of pilot studies: Recommendations for good practice: Design and analysis of pilot studies. *Journal of Evaluation in Clinical Practice,**10*, 307–312. 10.1111/j.2002.384.doc.x 15189396 10.1111/j..2002.384.doc.x

[23] Evans, C., Mellor-Clark, J., Margison, F., Barkham, M., Audin, K., Connell, J., & McGrath, G. (2000). CORE: Clinical outcomes in routine evaluation. *Journal of Mental Health,**9*(3), 247–255. 10.1080/jmh.9.3.247.255

[24] Mavranezouli, I., Brazier, J. E., Young, T. A., & Barkham, M. (2011). Using Rasch analysis to form plausible health states amenable to valuation: The development of CORE-6D from a measure of common mental health problems (CORE-OM). *Quality of Life Research,**20*(3), 321–333. 10.1007/s11136-010-9768-4 20972629 10.1007/s11136-010-9768-4

[25] Dixon, P. M., Saint-Maurice, P. F., Kim, Y., Hibbing, P., Bai, Y., & Welk, G. J. (2018). A primer on the use of equivalence testing for evaluating measurement agreement. *Medicine and Science in Sports and Exercise,**50*(4), 837–845. 10.1249/MSS.0000000000001481 29135817 10.1249/MSS.0000000000001481 PMC5856600

[26] Walker, E., & Nowacki, A. S. (2011). Understanding equivalence and noninferiority testing. *Journal of General Internal Medicine,**26*(2), 192–196. 10.1007/s11606-010-1513-8 20857339 10.1007/s11606-010-1513-8 PMC3019319

[27] Jones, B., & Kenward, M. G. (2014). *Design and analysis of cross-over trials* (3rd ed.). Chapman and Hall/CRC.

[28] Wang, Y., Tan, N. C., Tay, E. G., Thumboo, J., & Luo, N. (2015). Cross-cultural measurement equivalence of the 5-level EQ-5D (EQ-5D-5L) in patients with type 2 diabetes mellitus in Singapore. *Health and Quality of Life Outcomes,**13*, 103. 10.1186/s12955-015-0297-2 26179285 10.1186/s12955-015-0297-2 PMC4502594

[29] Machin, D., Campbell, M. J., Tan, S. B., & Tan, S. H. (2009). *Sample size tables for clinical studies* (3rd ed.). Wiley-Blackwell.

[30] Kim, H., Sefcik, J. S., & Bradway, C. (2017). Characteristics of qualitative descriptive studies: A systematic review. *Research in Nursing & Health,**40*(1), 23–42. 10.1002/nur.21768 27686751 10.1002/nur.21768 PMC5225027

[31] Neergaard, M. A., Olesen, F., Andersen, R. S., & Sondergaard, J. (2009). Qualitative description—The poor cousin of health research? *BMC Medical Research Methodology,**9*, 52. 10.1186/1471-2288-9-52 19607668 10.1186/1471-2288-9-52 PMC2717117

[32] Atkinson, J., Denmark, T., Marshall, J., Mummery, C., & Woll, B. (2015). Detecting cognitive impairment and dementia in deaf people: The British Sign Language cognitive screening test. *Archives of Clinical Neuropsychology,**30*(7), 694–711. 10.1093/arclin/acv042 26245349 10.1093/arclin/acv042

