# Supplementary material for: The influence of mode of remote delivery on health-related quality of life outcome measures in British Sign Language: a mixed methods pilot randomised crossover trial
Source: Qual Life Res. 2024 Dec 11;34(3):657–67. doi: 10.1007/s11136-024-03864-0 (PMC11919977; doi:10.1007/s11136-024-03864-0)
Supplement: Supplementary file 2 — Supplementary file2 (DOCX 27 KB) [file 11136_2024_3864_MOESM2_ESM.docx]

**Supplementary table 1.**

**Demographic information of the interviewed participants**

|  |  | n (%) |
| --- | --- | --- |
| Age in years  (Mean age: 44) | 30-39 | 3 (37.5%) |
|  | 40-49 | 3 (37.5%) |
|  | 50-64 | 2 (25%) |
|  | +65 | 0 (0%) |
| Gender identity | Female | 3 (37.5%) |
|  | Male | 4 (50%) |
|  | Transwoman | 1 (12.5%) |
| Ethnicity | Asian/Asian British Chinese | 0 (0%) |
|  | Asian/Asian British Pakistani | 0 (0%) |
|  | Mixed/Multiple ethnic group: Black Caribbean and White | 0 (0%) |
|  | Mixed/Multiple group: Any other | 0 (0%) |
|  | White: English/Welsh/Scottish/Northern Irish/British | 8 (100%) |
| Sexuality | Gay man | 3 (37.5%) |
|  | Gay woman/lesbian | 0 (0%) |
|  | Straight/heterosexual | 4 (50%) |
|  | Pansexual | 1 (12.5%) |
|  | Prefer not to say | 0 (0%) |
| Consider disabled | Yes | 8 (100%) |
|  | No | 0 (0%) |
|  | Prefer not to say | 0 (0%) |
| Employment status | Yes | 4 (50%) |
|  | No | 4 (50%) |
| Currently experiencing mental health difficulties | Yes | 3 (37.5%) |
|  | No | 3 (37.5%) |
|  | I don’t know | 2 (25%) |
|  | Missing answer | 0 (0%) |
